# TBAJ-587, a novel diarylquinoline, is active against *Mycobacterium abscessus*

**DOI:** 10.1128/aac.00945-24

**Published:** 2024-10-29

**Authors:** Junsheng Fan, Zhili Tan, Siyuan He, Anqi Li, Yaping Jia, Juan Li, Zhemin Zhang, Bing Li, Haiqing Chu

**Affiliations:** 1 Department of Respiratory and Critical Care Medicine, Shanghai Pulmonary Hospital, School of Medicine, Tongji University, Shanghai, China; 2 School of Medicine, Tongji University, Shanghai, China; 3 Shanghai Key Laboratory of Tuberculosis, Shanghai Pulmonary Hospital, School of Medicine, Tongji University, Shanghai, China; St. George's, University of London, London, United Kingdom

**Keywords:** TBAJ-587, diarylquinoline, nontuberculous mycobacteria, *Mycobacterium abscessus*

## Abstract

Nontuberculous mycobacteria (NTM) infections are extremely difficult to treat due to a natural resistance to many antimicrobials. TBAJ-587 is a novel diarylquinoline, which shows higher anti-tuberculosis activity, lower lipophilicity, and weaker inhibition of hERG channels than bedaquiline (BDQ). The susceptibilities of 11 NTM reference strains and 194 clinical *Mycobacterium abscessus* isolates to TBAJ-587 were determined by the broth microdilution assay. The activity of TBAJ-587 toward the growth of *M. abscessus* in macrophages was also evaluated. Minimum bactericidal concentration and time-kill kinetic assays were conducted to distinguish between the bactericidal and bacteriostatic activities of TBAJ-587. The synergy between TBAJ-587 and eight clinically important antibiotics was determined using a checkerboard assay. TBAJ-587 was highly effective against *M. abscessus* by targeting its F-ATP synthase *c* chain. The antimicrobial activities of TBAJ-587 and BDQ toward intracellular *M. abscessus* were comparable. The *in vivo* activities of TBAJ-587 and BDQ in an immunocompromised mouse model were also comparable. TBAJ-587 expressed bactericidal activity and was compatible with eight anti-NTM drugs commonly used in clinical practice; no antagonism was discovered. As such, TBAJ-587 represents a potential candidate for the treatment of NTM infections.

## INTRODUCTION

Nontuberculous mycobacteria (NTM) refer to mycobacteria other than *Mycobacterium tuberculosis* and *Mycobacterium leprae* (1). The incidence of NTM infections is growing rapidly; treatment is extremely difficult (2). Therapeutic regimens often contain a cocktail of several antimicrobials; treatment, which lasts for several months, can be unsatisfactory (1). New drugs are needed.

Bedaquiline (BDQ), the first-in-class diarylquinoline, is effective in treating nontuberculous mycobacterial lung diseases (3). TBAJ-587 and TBAJ-876, novel diarylquinolines first reported by Sutherland et al., just completed their phase 1 clinical trials (NCT04890535 and NCT04493671, respectively) (4). They have the same antimicrobial mechanism as BDQ but exhibit a weaker inhibition of the human ether-a-go-go-related gene (hERG) potassium channel and higher anti-tuberculosis activity (4–6). The minimal inhibitory concentration (MIC) of TBAJ-587 for *M. tuberculosis* H37Rv is 0.016 mg/L (5). *In vivo* experiments showed that TBAJ-587, alone or in combination with other antimicrobials, exhibited higher anti-tuberculosis activity than BDQ (6). Reportedly, TBAJ-876 is active against *Mycobacterium abscessus* (7). The activity of TBAJ-587 toward NTM, however, has not been reported.

Here, the antimicrobial activity of TBAJ-587 against *M. abscessus* was evaluated. TBAJ-587 was highly effective against *M. abscessus in vitro*. TBAJ-587 was also active against clinical *M. abscessus* isolates with Mab_2299c or Mab_4384 missense mutations. TBAJ-587 and BDQ were comparable in their abilities to inhibit the intracellular growth of *M. abscessus* in macrophages. The *in vivo* antibacterial activities of TBAJ-587 and BDQ in an immunocompromised mouse model were also similar. TBAJ-587 exhibited bactericidal activity and did not antagonize the activities of antibiotics frequently used to treat NTM infections. Spontaneous resistant mutation assays and molecular docking results indicate that TBAJ-587 targets the *M. abscessus* F-ATP synthase *c* chain. Consequently, TBAJ-587 potentially provides an additional approach to treating *M. abscessus* infections.

## RESULTS

### TBAJ-587 is active against NTM

A total of 11 NTM reference strains and 194 clinical *M. abscessus* isolates were collected. The MICs of TBAJ-587 for fast-growing and slow-growing NTM reference strains, except *Mycobacterium smegmatis*, were equal to or less than the MICs of BDQ (Table 1). The MICs of TBAJ-587 against rapidly growing clinical *M. abscessus* subsp. *abscessus* and *M. abscessus* subsp. *massiliense* isolates ranged from 0.002 to 0.5 mg/L and 0.0039 to 0.25 mg/L, respectively (Table 2). The MIC_50_ and MIC_90_ were the same for both subspecies. The MICs of TBAJ-587 affecting clinical *M. abscessus* isolates were significantly lower than the MICs of BDQ, imipenem, and clarithromycin (Wilcoxon signed rank test, *P* < 0.01; Table S1). The MICs of a third diarylquinoline, TBAJ-876, against clinical *M. abscessus* isolates were lower than TBAJ-587 (Wilcoxon signed rank test, *P* < 0.01; Table S1).

**TABLE 1:** MICs of TBAJ-587, TBAJ-876, and BDQ for NTM reference strains (mg/L)

	TBAJ-587	TBAJ-876	BDQ
Rapid-growing mycobacteria			
*M. abscessus* ATCC 19977	0.031	0.031	0.125
*M. massiliense* CIP 108297	0.031	0.031	0.125
*M. smegmatis* ATCC 607	0.004	≤0.002	≤0.002
*M. fortuitum* ATCC 35855	0.008	0.008	0.016
*M. peregrinum* ATCC 700686	≤0.002	≤0.002	≤0.002
Slow-growing mycobacteria			
*M. avium* ATCC 25291	≤0.002	≤0.002	≤0.002
*M. intracellulare* ATCC 13950	≤0.002	≤0.002	≤0.002
*M. kansasii* ATCC 12478	≤0.002	≤0.002	≤0.002
*M. gordonae* ATCC 14470	≤0.002	≤0.002	≤0.002
*M. szulgai* ATCC 35799	≤0.002	≤0.002	≤0.002
*M. scrofulaceum* ATCC 19981	0.008	≤0.002	0.016

**TABLE 2:** MICs of TBAJ-587, TBAJ-876, and BDQ for clinical *M. abscessus* isolates

Subspecies	Number of strains by MIC (mg/L)										MIC_50_ (mg/L)	MIC_90_ (mg/L)
	≤0.002	0.0039	0.0078	0.0156	0.0312	0.0625	0.125	0.25	0.5	4		
*abscessus* (*n* = 148)												
TBAJ-587	7	8	6	14	35	45	30	2	1	0	0.0625	0.125
TBAJ-876	5	3	20	10	49	52	9	0	0	0	0.0312	0.0625
BDQ	0	0	6	2	26	34	53	26	1	0	0.125	0.25
*massiliense* (*n* = 46)												
TBAJ-587	0	4	2	6	7	12	11	4	0	0	0.0625	0.125
TBAJ-876	1	1	1	7	16	17	3	0	0	0	0.0312	0.0625
BDQ	0	0	1	1	6	9	22	6	0	1	0.125	0.25

### TBAJ-587 expresses bactericidal activity

**Fig 1:**
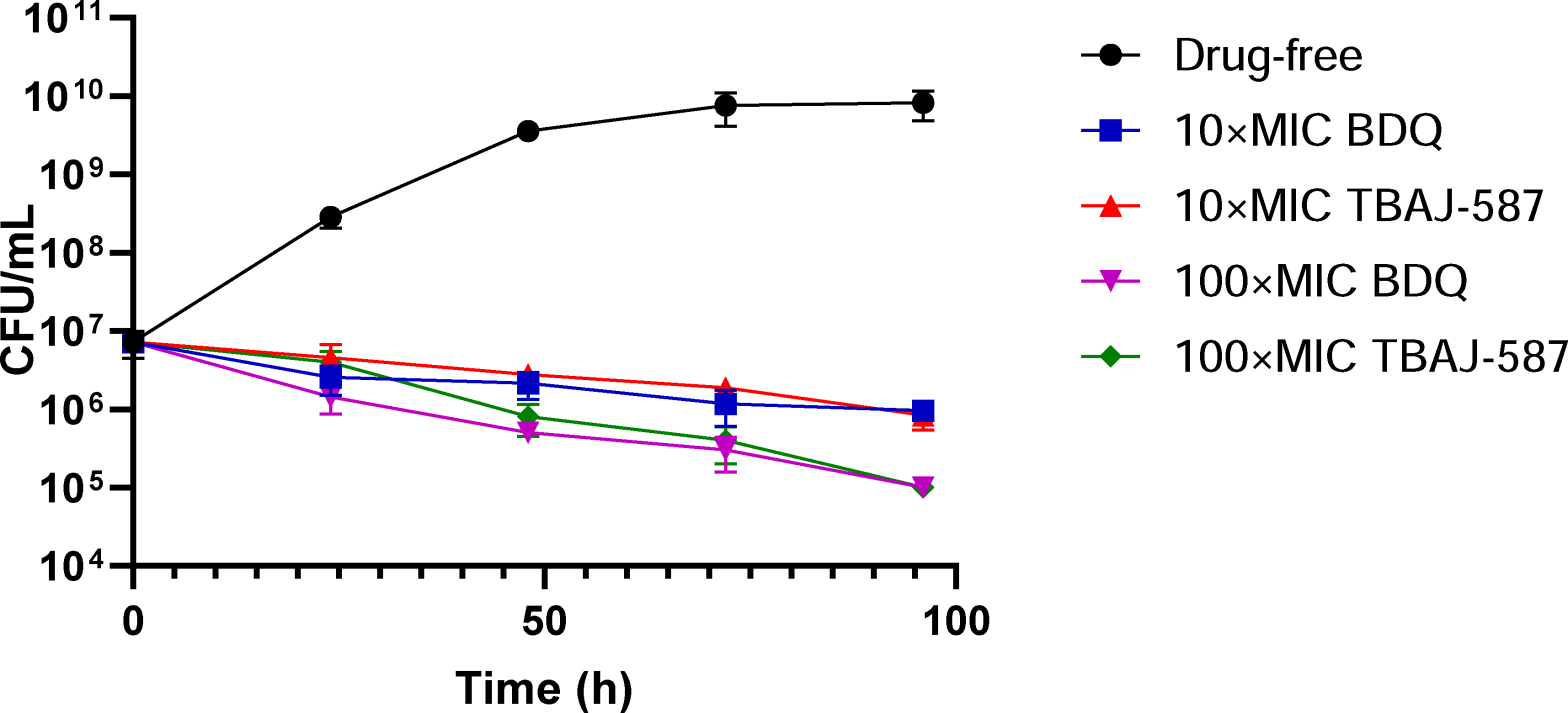
Antibacterial activities of TBAJ-587 and BDQ against *M. abscessus* reference strain ATCC 19977 growing extracellularly in Middlebrook 7H9+OADC medium. Values are the means ± SD of ≥3 independent experiments. All drug-treated values are significantly less than the inoculum and the drug-free values assessed at 24, 48, 72, and 96 hours post-inoculation, *P* < 0.05 (Mann-Whitney U-test).

The minimal bactericidal concentrations (MBC) of TBAJ-587 and BDQ for both *M. abscessus* and *Mycobacterium massiliense* were >1 mg/L and >4 mg/L, respectively, resulting in an MBC/MIC ratio >32. Time-kill kinetic assays were conducted as described previously using the *M. abscessus* reference strain ATCC 19977 (7, 8). Both 10- and 100-times the MICs of TBAJ-587 and BDQ inhibited the growth of *M. abscessus* (Fig. 1). The colony-forming units (CFUs) of each treated group were significantly lower at 24, 48, 72, and 96 hours post-inoculation than those of the drug-free control group, as well as the inoculum. These results demonstrate the bactericidal activity of both TBAJ-587 and BDQ on the extracellular growth of *M. abscessus in vitro*.

### TBAJ-587 is compatible with anti-NTM drugs frequently used clinically

Four NTM reference strains were selected to investigate the interaction between TBAJ-587 and eight clinically relevant anti-NTM drugs. No antagonism between TBAJ-587 and any of the other drugs was observed; indifference [fractional inhibitory concentration index (FICI) = 0.75–2] was the most common interaction observed (Table S2).

### TBAJ-587 and BDQ exhibit comparable intracellular antimicrobial activities

**Fig 2:**
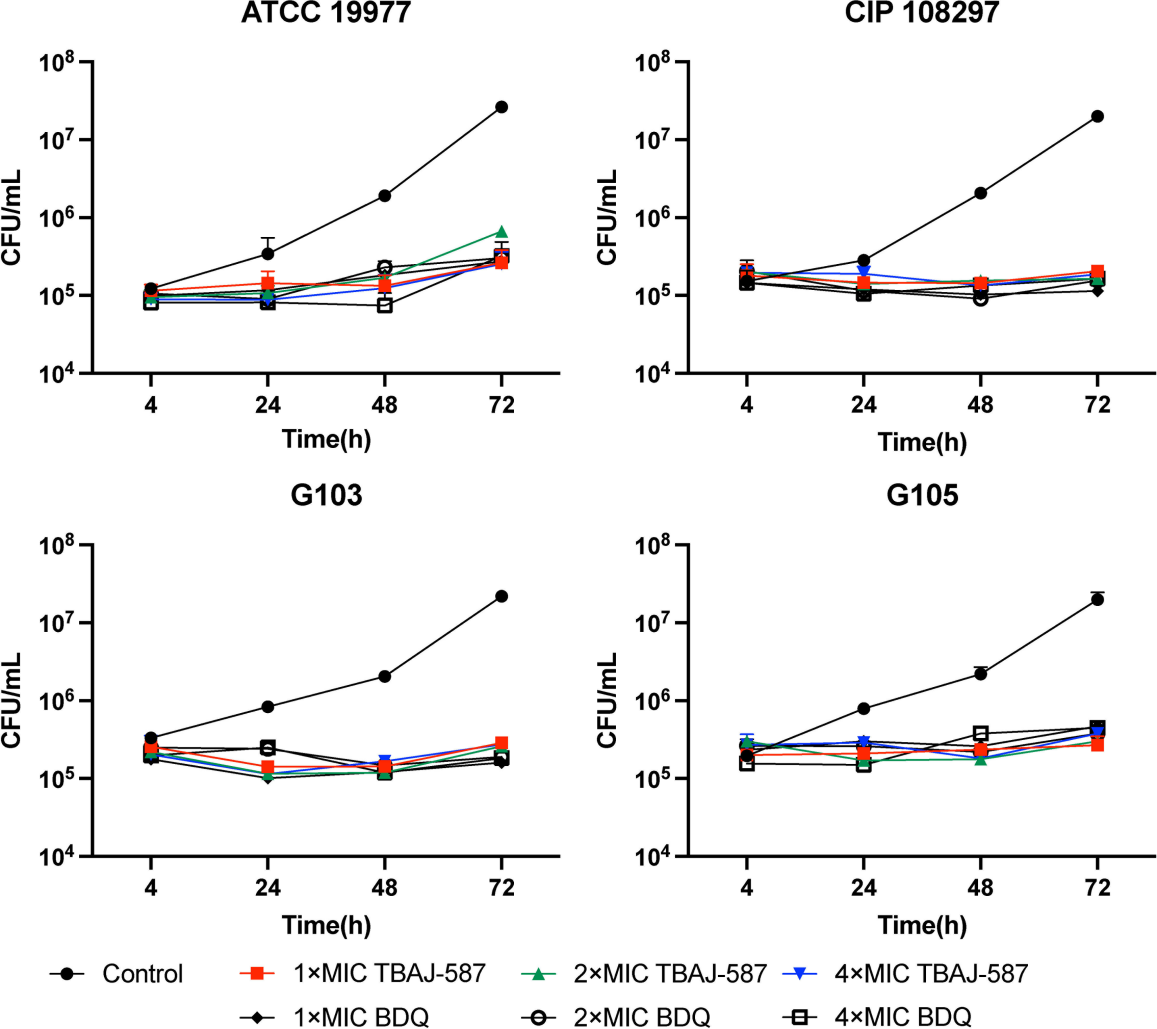
Relative intracellular antibacterial activities of TBAJ-587 and BDQ. Values are the means ± SD of ≥3 independent experiments. ATCC 19977, *M. abscessus* subsp. *abscessus* reference strain; CIP 108297, *M. abscessus* subsp. *massiliense* reference strain. G103, subsp. *abscessus* clinical isolate. G105, subsp. *massiliense* clinical isolate. All drug-treated and drug-free values determined at 48 and 72 hours are significantly different, *P* < 0.05 (Mann-Whitney U-test).

The intracellular antimicrobial activity of TBAJ-587 was determined by assessing its effects on two reference strains and two clinical *M. abscessus* isolates replicating in THP-1 macrophages. Both TBAJ-587 and BDQ inhibited the intracellular growth of *M. abscessus* assessed at 48 and 72 hours (Fig. 2). The intracellular antimicrobial activities of the drugs at 1×, 2×, and 4× MIC appeared comparable indicating that the intracellular activities of both TBAJ-587 and BDQ are high.

### TBAJ-587 is active against *M. abscessus* in a mouse model of lung infection

**Fig 3:**
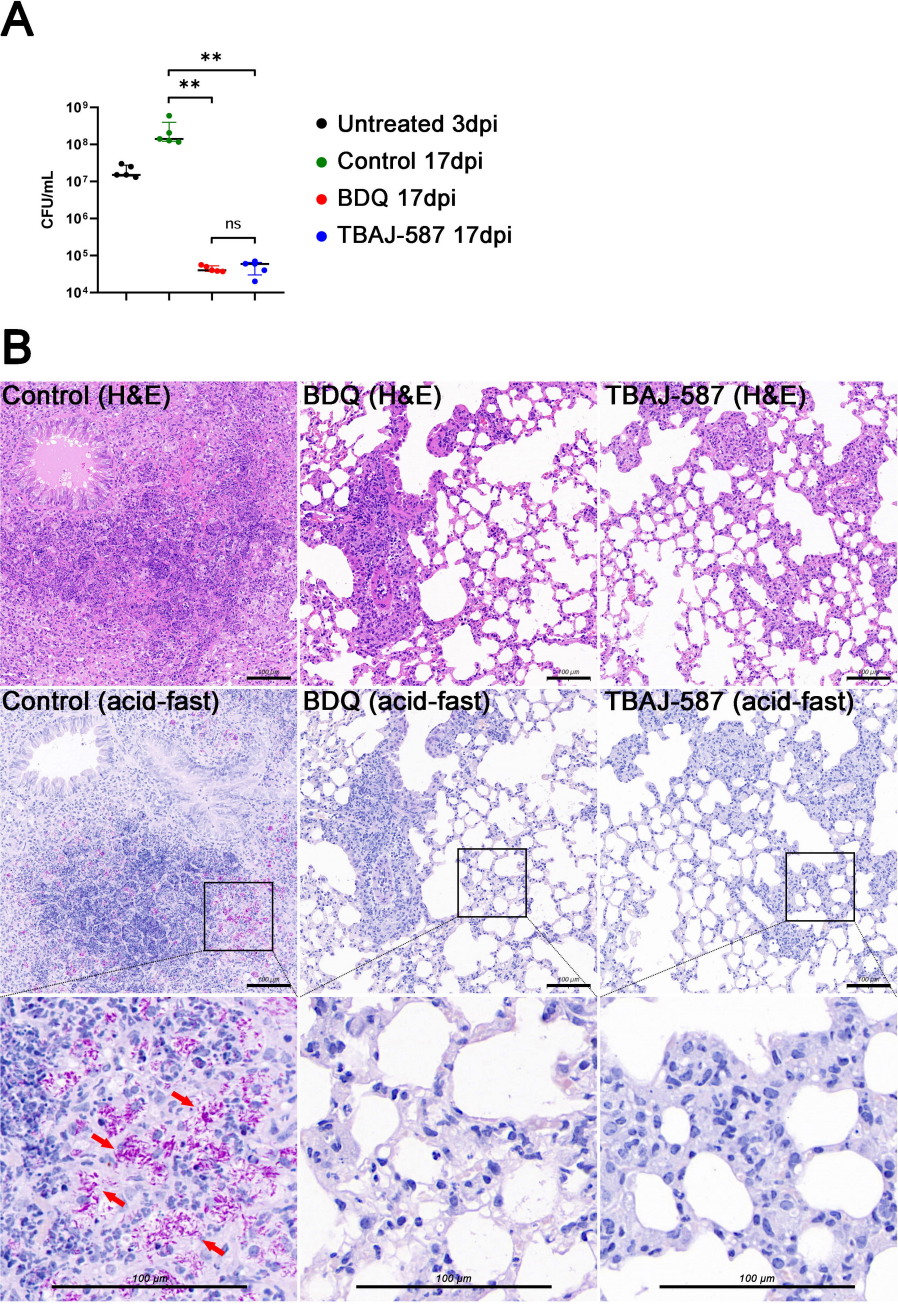
Antibacterial activities of BDQ and TBAJ-587 in a mouse model of *M. abscessus* lung infection. The control group was treated with solvent. (**A**) Lung tissue CFUs of each group. BDQ, bedaquiline; dpi, days post-infection. The error bars represent the median and interquartile range of each group. ns, no significance; significantly different: **, *P* < 0.01 (Mann-Whitney U-test). (**B**) Histopathological images of each treatment group. The top row is the images of hematoxylin and eosin (H&E) staining. The middle and bottom rows are the images of acid-fast staining; acid-fast bacilli are stained magenta. Scale bar, 100 µm.

Treatment with either TBAJ-587 or BDQ significantly reduced the bacterial load in the lungs of mice in a model of pulmonary infection compared to control mice treated with antibiotic solvent (Fig. 3A). Multifocal consolidation, significant neutrophil infiltration, and erythrocyte diapedesis were found in hematoxylin and eosin-stained lung sections derived from the solvent treated group (Fig. 3B). Profuse acid-fast bacilli were also found. These pathological findings were alleviated, however, by TBAJ-587 or BDQ treatment.

### TBAJ-587 targets the *M. abscessus* F-ATP synthase *c* chain

To determine whether TBAJ-587 exhibits the same mechanism of action against *M. abscessus* and *M. tuberculosis*, spontaneous TBAJ-587-resistant *M. abscessus* mutants were selected. The frequency of a spontaneous resistance mutation was approximately 1.4 × 10^−8^/CFU based on three independent experiments. Targeted sequencing of these mutants revealed that they all possessed a missense mutation in the *atpE* gene (*Mab_1448*) (Table 3). The MICs of both TBAJ-587 and BDQ for these isolates increased significantly compared with the MICs for their parent strain (*M. abscessus* ATCC 19977).

**TABLE 3:** Spontaneous *M. abscessus* TBAJ-587-resistant mutants and their susceptibilities to BDQ and TBAJ-587

Strain	Nucleotide changes in *atpE* gene	Amino acid changes in ATP synthase *C* chain	MIC (mg/L)^*a*^	
			BDQ	TBAJ-587
RM1	86A>C	D29A	>16	>16
RM2	86A>C	D29A	>16	>16
RM3	86A>C	D29A	>16	>16
RM4	86A>C	D29A	>16	>16
RM5	86A>C	D29A	>16	>16
RM6	86A>C	D29A	>16	>16
RM7	86A>C	D29A	>16	>16
RM8	86A>C	D29A	>16	>16
RM9	186G>T	E62D	>16	>16
RM10	86A>C	D29A	>16	>16

^*a*^ >16 indicates an MIC value that was greater than the highest TBAJ-587 or BDQ concentration tested.

**Fig 4:**
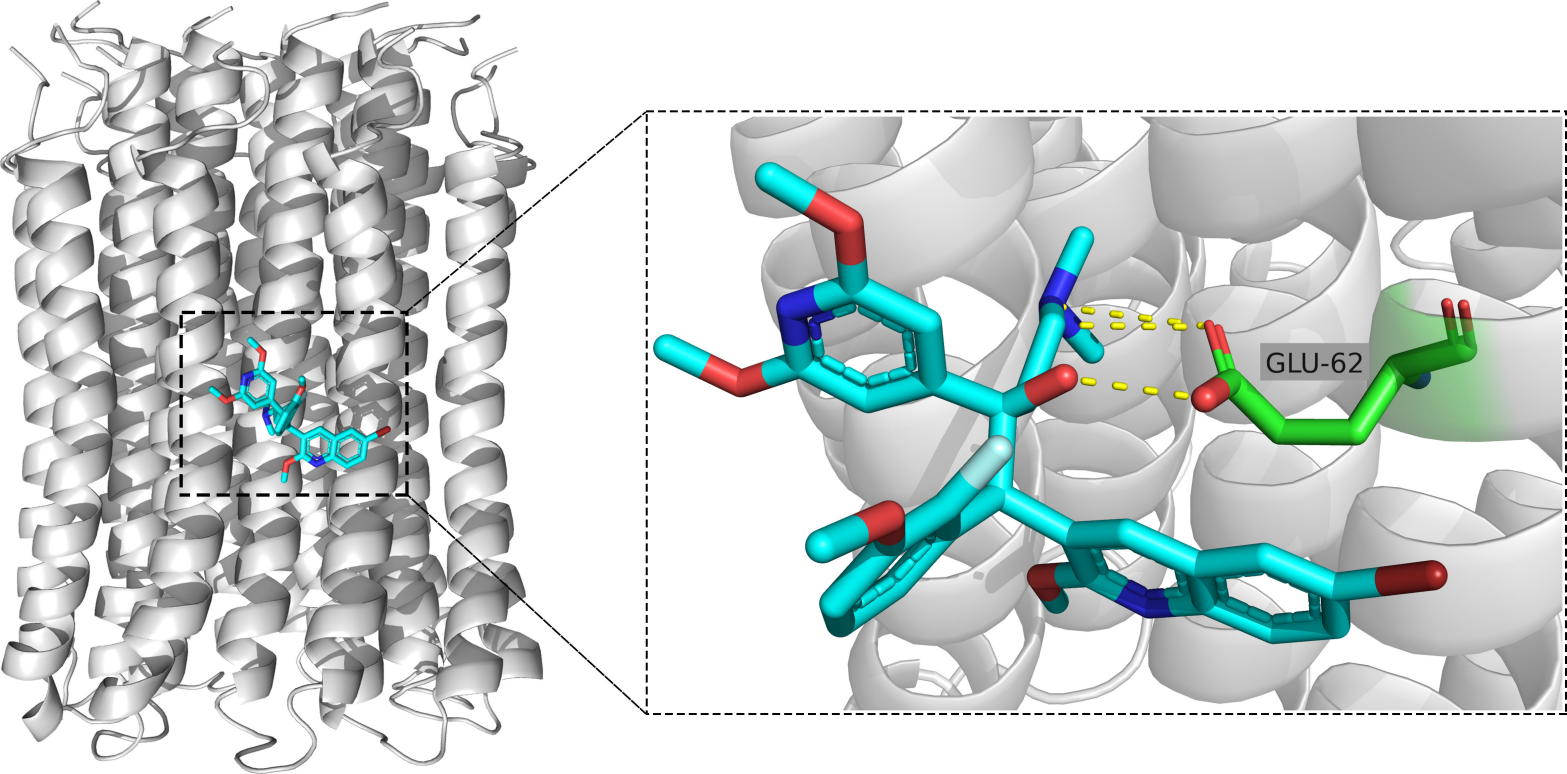
Molecular docking of TBAJ-587 (cyan) with *M. abscessus* ATP synthase C ring (gray). Glu^62^ of *M. abscessus* ATP synthase *c* subunit is green. Oxygen, nitrogen, and bromine atoms are red, blue, and maroon, respectively. The hydrogen bonds and salt bridge are represented by yellow dashed lines.

Multiple amino acid sequence alignment suggested that the *c* subunits of ATP synthase were highly conserved in these mycobacteria species. The amino acid sequence from Ala^8^ to Pro^80^ in the *c* subunit of *M. abscessus* corresponds to Ala^7^ to Pro^79^ sequence in its *M. tuberculosis* homolog (Fig. S1). Molecular docking results indicated that a salt bridge is formed between the dimethylamino group of TBAJ-587 and the carboxyl group of Glu^62^, a hydrogen bond was formed between the hydroxyl group of TBAJ-587 and the carboxyl group of Glu^62^ (Fig. 4). Taken together, these results suggest that the mechanism of TBAJ-587 action against *M. abscessus* and *M. tuberculosis* are the same, i.e., TBAJ-587 targets the *c* subunit of mycobacterial ATP synthase (9).

### TBAJ-587 is active against clinical *M. abscessus* isolates with Mab_2299c or Mab_4384 missense mutations

No *atpE* mutations were found among the 194 clinical *M. abscessus* isolates used in this study. Five isolates possessed missense mutations in Mab_2299c (Table S1). Importantly, the MICs of TBAJ-587 for these five isolates were significantly higher than for Mab_2299c wild-type isolates (Mann-Whitney U-test, *P* < 0.01). The MICs of BDQ for these five isolates also appeared to be higher although the increase was not statistically significant (Mann-Whitney U-test, *P* = 0.0653). Mab_4384 mutations were polymorphic, gene deletions were frequently discovered. No distinct differences in MICs were found between separate Mab_4384 genotypes. The MICs of TBAJ-587 for these Mab_2299c or Mab_4384 mutants were lower than 1 mg/L, however, indicating that TBAJ-587 was still active.

## DISCUSSION

NTM infections, which are notoriously difficult to treat, are a global concern (1). The present study compared the extracellular and intracellular antibacterial activities of TBAJ-587 and BDQ against *M. abscessus*.

BDQ, the first-in-class diarylquinoline anti-tuberculosis drug, acts by binding the *c*-ring of mycobacterial F-ATP synthase, which results in the depletion of intracellular ATP (10). Xu et al. reported that TBAJ-587 was more effective than BDQ in treating *M. tuberculosis* H37Rv infections both *in vivo* and *in vitro* (5). To our knowledge, the activity of TBAJ-587 against NTM has not been reported. In the present study, TBAJ-587 showed considerable activity against *M. abscessus in vitro*. The MICs of TBAJ-587 for clinical *M. abscessus* isolates were significantly lower than those of BDQ, imipenem, and clarithromycin (Table S1). The antibacterial activities of TBAJ-587 and BDQ against *M. abscessus* growing intracellularly in macrophages *in vitro* or in a mouse model of lung infection *in vivo* were also similar though the MICs of TBAJ-587 exhibited a lower trend.

TBAJ-876 is also a 3,5-dialkoxypyridine analog of BDQ (4). Sarathy et al. reported that TBAJ-876 is active against *M. abscessus*; the MIC for *M. abscessus* ATCC 19977 was 1.05 µM (0.69 mg/L) (7). In the present study, the MICs of both TBAJ-587 and TBAJ-876 for *M. abscessus* ATCC 19977 were 0.03125 mg/L (Table 1). However, TBAJ-876 appeared to be more effective than TBAJ-587 against clinical *M. abscessus* isolates *in vitro* (Table 2).

Xu et al. reported that TBAJ-587 was active against a resistant *M. tuberculosis Rv0678* mutant (5). Missense mutations in *Rv0678* can lead to the upregulation of MmpS5-MmpL5 efflux pumps, resulting in a moderate level of BDQ resistance (11). There is no homolog of Rv0678 in the *M. abscessus* genome (12, 13). Rather, some TetR family transcription repressors (Mab_2299c and Mab_4384) along with their adjacent *mmpS* and *mmpL* genes are reported to mediate drug efflux and BDQ resistance (12–16). The present study showed that TBAJ-587 was still active against clinical *M. abscessus* isolates that harbor Mab_2299c or Mab_4384 missense mutations.

MBC assays determined that the MBC/MIC ratios of TBAJ-587 and BDQ were greater than 32. Nevertheless, the time-kill kinetic curves showed that the CFUs of each treatment group assessed between 24 and 96 hours post-inoculation were significantly lower than the original inoculum indicating that both TBAJ-587 and BDQ were bactericidal. Notably, however, 10× and 100× MICs of either drug failed to achieve a 3 log_10_ decrease in CFU within 96 hours compared to the inoculum (Fig. 1). This could explain the failure of TBAJ-587 and BDQ at 32× MIC to eradicate 99.9% of the final inoculum in MBC determination assays. Considering the results of both the MBC and time-kill kinetic assays, TBAJ-587 and BDQ were judged to be bactericidal against *M. abscessus in vitro*. These findings correlate with previous studies reporting that both BDQ and TBAJ-876 exerted bactericidal effects on *M. tuberculosis* and *M. smegmatis* (17, 18).

A spontaneous resistant mutation assay revealed that resistant isolates selected on 7H10+OADC agar plates containing 1.25 mg/L TBAJ-587 possessed either a D29A or an E62D mutation in the F-ATP synthase *c* subunit encoded by *atpE*. These resistant mutations resulted in a >512-fold increase in the MIC of TBAJ-587. The mutants were also cross-resistant to BDQ. Amino acid sequence alignment suggested that Asp^29^ and Glu^62^ of the *M. abscessus* ATP synthase *c* subunit correspond to Asp^28^ and Glu^61^ of its *M. tuberculosis* homolog, respectively (Fig. S1). Therefore, the *M. abscessus* D29A and E62D mutations correspond to the D28A and E61D mutations which were reported previously in spontaneous, diarylquinoline-resistant *M. tuberculosis* mutants (19, 20).

One limitation of the current study lies in that all of the clinical *M. abscessus* isolates were obtained from a single center, i.e., Shanghai Pulmonary Hospital. Therefore, the genetic diversities between these clinical isolates may be limited.

In conclusion, TBAJ-587 expressed high antimicrobial activity against *M. abscessus*. It was compatible with anti-NTM drugs that frequently comprise therapeutic cocktails used to treat NTM infections; no antagonism occurred. As such, TBAJ-587 represents a potential candidate to include in *M. abscessus* treatment regimes.

## MATERIALS AND METHODS

### Strains and growth conditions

Eleven NTM reference strains enumerated in Table 1 were purchased from the American Type Culture Collection (Manassas, VA, USA) and the Biological Resource Center of Institute Pasteur (Paris, France) as described previously (8). One hundred ninety-four clinical *M. abscessus* isolates used in this study were obtained from Shanghai Pulmonary Hospital as described previously (8). All mycobacteria strains were cultured in Middlebrook 7H9 broth (Becton, Dickinson and Company, Franklin Lakes, NJ, USA) or on Middlebrook 7H10 agar (Becton, Dickinson) supplemented with 10% OADC enrichment.

### Antimicrobial agents, MIC, and MBC determination

TBAJ-587, BDQ, clarithromycin, imipenem, amikacin, rifabutin, linezolid, clofazimine, moxifloxacin, and tigecycline were purchased from MedChemExpress (Monmouth Junction, NJ, USA). TBAJ-876 was synthesized by BioDuro (Beijing, China) as described previously (7). All the antimicrobial agents were dissolved in absolute dimethyl sulfoxide at a concentration of 1,024 mg/L prior to MIC determination assays. The MICs of TBAJ-587, TBAJ-876, and BDQ against NTM reference strains and clinical isolates were determined by broth microdilution analysis according to the Clinical and Laboratory Standards Institute guidelines M24-A2 (21). *M. abscessus* reference strains ATCC 19977 and CIP 108297 were used to determine the MBC. After the MIC values were recorded, the contents of each well in which the drug concentrations were greater than MIC were resuspended, serially diluted, and spread on Middlebrook 7H10 agar plates supplemented with 10% OADC enrichment. The plates were incubated at 37°C for another 3 days before the CFUs were counted. The MBC of an antimicrobial for a particular strain was defined as the minimum concentration that inhibited 99.9% bacterial growth (22, 23).

### Intracellular antibacterial activity assay

Macrophages derived from the human monocyte cell line, THP-1 (purchased from Cell Bank/Stem Cell Bank, Chinese Academy of Sciences. Shanghai, China), were infected with *M. abscessus* reference strain ATCC 19977, *M. massiliense* reference strain CIP 108297, and clinical isolates *M. abscessus* G103 (subsp. *abscessus*) or G105 (subsp. *massiliense*) at a multiplicity of infection = 10 as described previously (24). Cell lysates, prepared 4, 24, 48, and 72 hours after the addition of TBAJ-587 or BDQ, were spread onto Middlebrook 7H10 agar plates supplemented with 10% OADC enrichment. The number of surviving intracellular *M. abscessus* was calculated from CFUs that grew (8, 25–27).

### Time-kill kinetic assay

*M. abscessus* ATCC 19977 was used to evaluate the extracellular killing kinetics of TBAJ-587 and BDQ. A mid-log phase culture was adjusted to 0.0125 OD_600_ using Middlebrook 7H9 broth supplemented with 10% OADC and 0.05% Tween-80. TBAJ-587 (0.3125 or 3.125 mg/L, final concentration) or BDQ (1.25 or 12.5 mg/L, final concentration) was added to the medium. Each group was then incubated for 4 days at 37°C on an environmental shaker rotating at 220 rpm. Aliquots of each group were serially diluted and spread onto Middlebrook 7H10 agar plates every 24 hours. The number of viable bacteria was calculated from the CFUs that grew.

### Drug synergy assay

The compatibility of TBAJ-587 with eight antimicrobials frequently used in clinical practice to treat NTM infections, i.e., clarithromycin, imipenem, amikacin, rifabutin, linezolid, clofazimine, moxifloxacin, and tigecycline, was evaluated using the checkerboard dilution test (28). Two rapid-growing mycobacteria (*M. abscessus* and *M. massiliense*) and two slow-growing mycobacteria (*Mycobacterium avium* and *Mycobacterium intracellulare*) reference strains were used to test drug compatibility. FICI was defined as the MIC of TBAJ-587 in combination/MIC of TBAJ-587 alone + MIC of the second drug in combination/MIC of the second drug alone. Two drugs were considered: synergistic if the FICI was ≤0.5, antagonistic if the FICI was >4, and indifferent if the FICI was >0.5 and ≤4, according to mycobacteria drug susceptibility studies published previously (8, 26).

### Animal model

Six-week-old male BALB/c mice were purchased from SPF Biotech (Beijing, China). Five mice/cages were housed in a dedicated vivarium (18°C–23°C ambient temperature, 40%–60% humidity, 14 hour:10 hour light cycle) with clean food, water, and bedding. All animal experiments were approved by the Ethics Committee of Shanghai Pulmonary Hospital (number of approved protocols: K24-309).

The mice were rendered neutropenic by intraperitoneal injection of 150 mg/kg cyclophosphamide on days 4 and 1 prior to infection as described previously (8, 26). Subsequently, the mice were infected intranasally with *M. abscessus* CIP 108297 (4 × 10^7^ CFUs/mouse). On day 3 post-infection, five mice were selected at random to evaluate the reliability of the infection model. The remaining 15 mice were allocated to three groups: group 1, the control group administered solvent (20% hydroxypropyl-β-cyclodextrin solution acidified with 1.5% 1 N HCl); group 2, treated daily for 14 days by oral gavage with 25 mg/kg TBAJ-587 dissolved in solvent; group 3, treated daily for 14 days by oral gavage with 25 mg/kg BDQ dissolved in solvent. After 14 days, the mice were euthanized and a pneumonectomy was performed. The right upper lobes were dissected for histopathological examination. Their remaining lung tissues were homogenized, serial diluted, and spread on Middlebrook 7H10 agar plates supplemented with 10% OADC for CFU determination.

### Isolation of spontaneous resistant mutants

*M. abscessus* ATCC 19977 colonies were grown in 10 mL Middlebrook 7H9 broth supplemented with 10% OADC to 1.0 OD_600_. A 5 mL culture aliquot was used as a control for sequencing. The culture broth that remained was centrifuged and the cell pellet was resuspended in 0.5 mL Middlebrook 7H9 broth. One hundred microliters of suspended bacteria were spread onto Middlebrook 7H10 agar plates supplemented with 10% OADC enrichment and 1.25 mg/L TBAJ-587. After incubation for 7 days at 37°C, the TBAJ-587-resistant colonies that grew were confirmed by MIC determination. Subsequently, Middlebrook 7H9 broth supplemented with 10% OADC was inoculated with these resistant colonies and their genomic DNA was extracted using HiPure Bacterial DNA Kit (Magen Biotechnology; Guangzhou, Guangdong, China). The *atpE* (*Mab_1448*) coding sequences of these TBAJ-587-resistant strains were Sanger sequenced using the following primers: (atpE-Forward: TGATCGCGATGTTCCCCGCA; atpE-Reverse: CGGTGAATTGCTCAGCGGCG).

### Bioinformatic analysis and molecular docking

The amino acid sequences of the ATP synthase *c* chain of mycobacteria strains were obtained from the National Center for Biotechnology Information (Bethesda, MD, USA). The secondary structure of *M. tuberculosis* ATP synthase *c* chain was obtained from the UniProt database (UniProt ID: B1MLV7) (https://www.uniprot.org/) (29). Multiple sequence alignment was performed using ClustalW (https://www.genome.jp/tools-bin/clustalw) (30). ESPript 3.0 (https://espript.ibcp.fr/) was used to visualize the results of multiple sequence alignment (31). A model of the *M. abscessus* ATP synthase C ring was built by homology modeling using PyMol; the crystal structure of *Mycobacterium phlei* ATP synthase C ring (PDB ID: 4V1G) was used as the template (32, 33). The structure of TBAJ-587 (PubChem ID: 138319677) was retrieved from the PubChem database (https://pubchem.ncbi.nlm.nih.gov/). TBAJ-587 was loaded in .sdf format and docked into *M. abscessus* ATP synthase C ring using Molecular Operating Environment software version 2022 (Chemical Computing Group; Montreal, Canada). The optimal docking result was selected based on the docking score and the rationality of protein-ligand interaction. The reference sequence of the *M. abscessus* ATCC 19977 genome (GenBank ID: CU458896.1) was obtained from the National Center for Biotechnology Information (Bethesda, MD, USA). The whole-genome sequences of the 194 clinical *M. abscessus* isolates, which were sequenced in previous studies, were surveyed (15, 34). The DNA sequences of *atpE*, *Mab_2299*c, and *Mab_4384* of these clinical *M. abscessus* isolates were aligned to the homologous sequences of the reference strain ATCC 19977 using SnapGene version 4.3.6 (Dotmatics; Boston, MA, USA).

### Statistical analysis

GraphPad Prism version 10.1.0 (Dotmatics; Boston, MA, USA) was used for all statistical analyses. Wilcoxon signed-rank test was used to compare the MIC differences between the two groups. Mann-Whitney U-test was used to compare differences in CFUs between the two groups. *P* < 0.05 was considered statistically significant.

## SUPPLEMENTAL MATERIAL

Supplemental material aac.00945-24-s0001.pdf Figure S1; Tables S1 and S2.

ASM does not own the copyrights to Supplemental Material that may be linked to, or accessed through, an article. The authors have granted ASM a non-exclusive, world-wide license to publish the Supplemental Material files. Please contact the corresponding author directly for reuse.

## References

[1] Griffith DE, Aksamit T, Brown-Elliott BA, Catanzaro A, Daley C, Gordin F, Holland SM, Horsburgh R, Huitt G, Iademarco MF, Iseman M, Olivier K, Ruoss S, von Reyn CF, Wallace RJ, Winthrop K, Infectious Disease Society of America, American Thoracic Society, Infectious Disease Society of America. 2007. An official ATS/IDSA statement: diagnosis, treatment, and prevention of nontuberculous mycobacterial diseases. Am J Respir Crit Care Med 175:367–416. doi:10.1164/rccm.200604-571ST 17277290

[2] Adjemian J, Olivier KN, Seitz AE, Holland SM, Prevots DR. 2012. Prevalence of nontuberculous mycobacterial lung disease in U.S. Medicare beneficiaries. Am J Respir Crit Care Med 185:881–886. doi:10.1164/rccm.201111-2016OC 22312016 PMC3360574

[3] Philley JV, Wallace RJ Jr, Benwill JL, Taskar V, Brown-Elliott BA, Thakkar F, Aksamit TR, Griffith DE. 2015. Preliminary results of bedaquiline as salvage therapy for patients with nontuberculous mycobacterial lung disease. Chest 148:499–506. doi:10.1378/chest.14-2764 25675393 PMC4694173

[4] Sutherland HS, Tong AST, Choi PJ, Blaser A, Conole D, Franzblau SG, Lotlikar MU, Cooper CB, Upton AM, Denny WA, Palmer BD. 2019. 3,5-dialkoxypyridine analogues of bedaquiline are potent antituberculosis agents with minimal inhibition of the hERG channel. Bioorg Med Chem 27:1292–1307. doi:10.1016/j.bmc.2019.02.026 30803745 PMC6467547

[5] Xu J, Converse PJ, Upton AM, Mdluli K, Fotouhi N, Nuermberger EL. 2021. Comparative efficacy of the novel diarylquinoline TBAJ-587 and bedaquiline against a resistant Rv0678 mutant in a mouse model of tuberculosis . Antimicrob Agents Chemother 65:e02418–20. doi:10.1128/AAC.02418-20 33526488 PMC8097419

[6] Li S-Y, Converse PJ, Betoudji F, Lee J, Mdluli K, Upton A, Fotouhi N, Nuermberger EL. 2023. Next-generation diarylquinolines improve sterilizing activity of regimens with pretomanid and the novel oxazolidinone TBI-223 in a mouse tuberculosis model. Antimicrob Agents Chemother 67:e0003523. doi:10.1128/aac.00035-23 36920217 PMC10112056

[7] Sarathy JP, Ganapathy US, Zimmerman MD, Dartois V, Gengenbacher M, Dick T. 2020. TBAJ-876, a 3,5-dialkoxypyridine analogue of bedaquiline, is active against Mycobacterium abscessus. Antimicrob Agents Chemother 64:e02404-19. doi:10.1128/AAC.02404-19 31964791 PMC7179298

[8] Wu W, He S, Li A, Guo Q, Tan Z, Liu S, Wang X, Zhang Z, Li B, Chu H. 2022. A novel leucyl-tRNA synthetase inhibitor, MRX-6038, expresses anti-Mycobacterium abscessus activity in vitro and in vivo. Antimicrob Agents Chemother 66:e0060122. doi:10.1128/aac.00601-22 35969055 PMC9487484

[9] Courbon GM, Palme PR, Mann L, Richter A, Imming P, Rubinstein JL. 2023. Mechanism of mycobacterial ATP synthase inhibition by squaramides and second generation diarylquinolines. EMBO J 42:e113687. doi:10.15252/embj.2023113687 37377118 PMC10390873

[10] Krah A, Grüber G, Bond PJ. 2022. Binding properties of the anti-TB drugs bedaquiline and TBAJ-876 to a mycobacterial F-ATP synthase. Curr Res Struct Biol 4:278–284. doi:10.1016/j.crstbi.2022.09.001 36186842 PMC9516385

[11] Ismail N, Ismail NA, Peters RPH. 2019. In vitro study of stepwise acquisition of rv0678 and atpE mutations conferring bedaquiline resistance. Antimicrob Agents Chemother 63:e00292–19. doi:10.1128/AAC.00292-19 31138569 PMC6658778

[12] Halloum I, Viljoen A, Khanna V, Craig D, Bouchier C, Brosch R, Coxon G, Kremer L. 2017. Resistance to thiacetazone derivatives active against Mycobacterium abscessus involves mutations in the MmpL5 transcriptional repressor MAB_4384. Antimicrob Agents Chemother 61:e02509-16. doi:10.1128/AAC.02509-16 28096157 PMC5365672

[13] Chen Y, Chen J, Zhang S, Shi W, Zhang W, Zhu M, Zhang Y. 2018. Novel mutations associated with clofazimine resistance in Mycobacterium abscessus. Antimicrob Agents Chemother 62:e00544-18. doi:10.1128/AAC.00544-18 29712660 PMC6021651

[14] Zhang R, Luo S, Wang N, Zhang H, Wu X. 2023. Epidemiology of nontuberculous mycobacteria in Nanjing and MAB_0540 mutations associated with clofazimine resistance in Mycobacterium abscessus. Infect Drug Resist 16:2751–2764. doi:10.2147/IDR.S408986 37180636 PMC10171220

[15] Li B, Ye M, Guo Q, Zhang Z, Yang S, Ma W, Yu F, Chu H. 2018. Determination of MIC distribution and mechanisms of decreased susceptibility to bedaquiline among clinical isolates of Mycobacterium abscessus. Antimicrob Agents Chemother 62:e00175-18. doi:10.1128/AAC.00175-18 29712658 PMC6021634

[16] Gutiérrez AV, Richard M, Roquet-Banères F, Viljoen A, Kremer L. 2019. The TetR family transcription factor MAB_2299c regulates the expression of two distinct MmpS-MmpL efflux pumps involved in cross-resistance to clofazimine and bedaquiline in Mycobacterium abscessus. Antimicrob Agents Chemother 63:e01000-19. doi:10.1128/AAC.01000-19 31332077 PMC6761555

[17] Sarathy JP, Ragunathan P, Cooper CB, Upton AM, Grüber G, Dick T. 2020. TBAJ-876 displays bedaquiline-like mycobactericidal potency without retaining the parental drug’s uncoupler activity. Antimicrob Agents Chemother 64:e01540-19. doi:10.1128/AAC.01540-19 PMC6985740 31712198

[18] Hards K, Robson JR, Berney M, Shaw L, Bald D, Koul A, Andries K, Cook GM. 2015. Bactericidal mode of action of bedaquiline. J Antimicrob Chemother 70:2028–2037. doi:10.1093/jac/dkv054 25754998

[19] Sarathy JP, Ragunathan P, Shin J, Cooper CB, Upton AM, Grüber G, Dick T. 2019. TBAJ-876 retains bedaquiline’s activity against subunits c and ε of Mycobacterium tuberculosis F-ATP synthase. Antimicrob Agents Chemother 63:e01191-19. doi:10.1128/AAC.01191-19 PMC6761534 31358589

[20] Segala E, Sougakoff W, Nevejans-Chauffour A, Jarlier V, Petrella S. 2012. New mutations in the mycobacterial ATP synthase: new insights into the binding of the diarylquinoline TMC207 to the ATP synthase C-ring structure. Antimicrob Agents Chemother 56:2326–2334. doi:10.1128/AAC.06154-11 22354303 PMC3346594

[21] Clinical and Laboratory Standards Institute. 2011. Susceptibility testing of mycobacteria, nocardiae, and other aerobic actinomycetes; approved standard. 2nd ed. Document M24-A2. Wayne, PA. 31339680

[22] Clinical and Laboratory Standards Institute. 1999. Methods for determining bactericidal activity of antimicrobial agents; approved guideline. Document M26-A. Wayne, PA.

[23] Maurer FP, Bruderer VL, Ritter C, Castelberg C, Bloemberg GV, Böttger EC. 2014. Lack of antimicrobial bactericidal activity in Mycobacterium abscessus. Antimicrob Agents Chemother 58:3828–3836. doi:10.1128/AAC.02448-14 24752273 PMC4068550

[24] Molina-Torres CA, Tamez-Peña L, Castro-Garza J, Ocampo-Candiani J, Vera-Cabrera L. 2018. Evaluation of the intracellular activity of drugs against Mycobacterium abscessus using a THP-1 macrophage model. J Microbiol Methods 148:29–32. doi:10.1016/j.mimet.2018.03.020 29626567

[25] Guo Q, Xu L, Tan F, Zhang Y, Fan J, Wang X, Zhang Z, Li B, Chu H. 2021. A novel oxazolidinone, contezolid (MRX-I), expresses anti-Mycobacterium abscessus activity in vitro. Antimicrob Agents Chemother 65:e0088921. doi:10.1128/AAC.00889-21 34460305 PMC8522767

[26] Zhang S, Zou Y, Guo Q, Chen J, Xu L, Wan X, Zhang Z, Li B, Chu H. 2020. AR-12 exhibits direct and host-targeted antibacterial activity toward Mycobacterium abscessus. Antimicrob Agents Chemother 64:e00236-20. doi:10.1128/AAC.00236-20 32482678 PMC7526805

[27] Olivença F, Pires D, Silveiro C, Gama B, Holtreman F, Anes E, Catalão MJ. 2024. Ethambutol and meropenem/clavulanate synergy promotes enhanced extracellular and intracellular killing of Mycobacterium tuberculosis. Antimicrob Agents Chemother 68:e0158623. doi:10.1128/aac.01586-23 38411952 PMC10989012

[28] Hsieh MH, Yu CM, Yu VL, Chow JW. 1993. Synergy assessed by checkerboard. A critical analysis. Diagn Microbiol Infect Dis 16:343–349. doi:10.1016/0732-8893(93)90087-n 8495592

[29] Consortium TU. 2021. UniProt: the universal protein knowledgebase in 2021. Nucleic Acids Res 49:D480–D489. doi:10.1093/nar/gkaa1100 33237286 PMC7778908

[30] Thompson JD, Higgins DG, Gibson TJ. 1994. CLUSTAL W: improving the sensitivity of progressive multiple sequence alignment through sequence weighting, position-specific gap penalties and weight matrix choice. Nucleic Acids Res 22:4673–4680. doi:10.1093/nar/22.22.4673 7984417 PMC308517

[31] Robert X, Gouet P. 2014. Deciphering key features in protein structures with the new ENDscript server. Nucleic Acids Res 42:W320–W324. doi:10.1093/nar/gku316 24753421 PMC4086106

[32] Preiss L, Langer JD, Yildiz Ö, Eckhardt-Strelau L, Guillemont JEG, Koul A, Meier T. 2015. Structure of the mycobacterial ATP synthase Fo rotor ring in complex with the anti-TB drug bedaquiline. Sci Adv 1:e1500106. doi:10.1126/sciadv.1500106 26601184 PMC4640650

[33] Webb B, Sali A. 2016. Comparative protein structure modeling using MODELLER. Curr Protoc Bioinformatics 54:5. doi:10.1002/cpbi.3 PMC5031415 27322406

[34] Li B, Yang S, Chu H, Zhang Z, Liu W, Luo L, Ma W, Xu X. 2017. Relationship between antibiotic susceptibility and genotype in Mycobacterium abscessus clinical isolates. Front Microbiol 8:1739. doi:10.3389/fmicb.2017.01739 28959242 PMC5603792

