# Supplementary material for: TBAJ-587, a novel diarylquinoline, is active against Mycobacterium abscessus
Source: Antimicrob Agents Chemother. 2024 Oct 29;68(12):e00945-24. doi: 10.1128/aac.00945-24 (PMC11619292; doi:10.1128/aac.00945-24)
Supplement: Supplemental material — Figure S1; Tables S1 and S2. [file aac.00945-24-s0001.pdf]

Supplementary Material

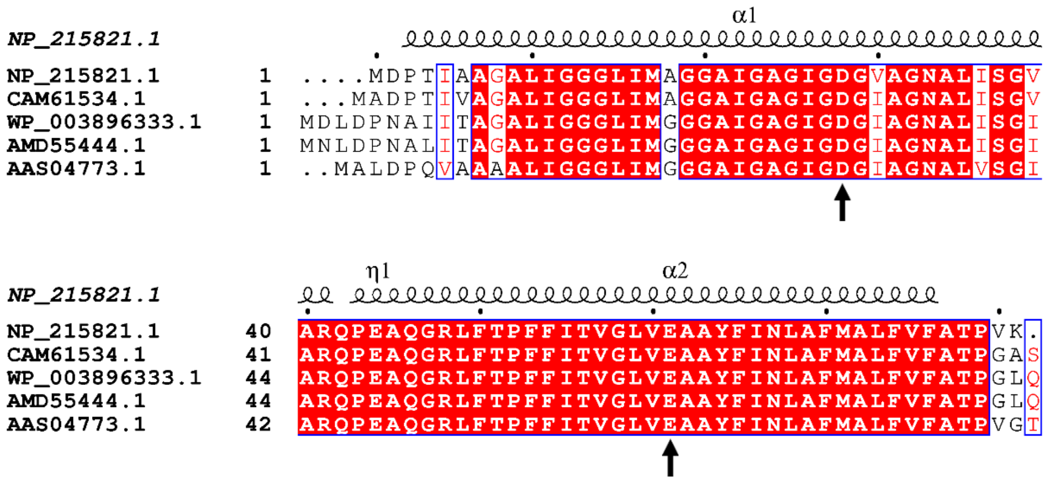

Supplementary Figure 1. Alignment of the ATP synthase *c* subunit amino acid sequences of: *M. tuberculosis* (GenBank ID: NP_215821.1), *M. abscessus* (CAM61534.1), *M. smegmatis* (WP_003896333.1), *M. fortuitum* (AMD55444.1) and *M. avium* (AAS04773.1). White amino acid residues on a red background are identical. Red residues on a white background are similar. Blue rectangles represent global similarity frames. The residues found to be involved in TBAJ-587 resistance (Asp^{29} and Glu^{62} of *M. abscessus* ATP synthase *c* subunit) are indicated by arrows.

Supplementary Table 1. Effects of MAB_2299c and MAB_4384 mutations on the MICs of BDQ, TBAJ-587 and TBAJ-876 for 194 clinical *M. abscessus* isolates.

Isolate	Subspecies	MAB_2299c mutation	MAB_4384 mutation	MICs (mg/L)				
				BDQ	TBAJ-587	TBAJ-876	Imipenem	Clarithromycin
A8	*abscessus*	-	DEL[a]	0.0156	0.0156	0.0078	4	0.0625
A10	*abscessus*	-	-	0.125	0.125	0.0625	8	0.5
A25	*abscessus*	-	Q216R	0.0078	0.125	0.0625	8	0.25
A35	*abscessus*	-	-	0.0625	0.125	0.0078	32	0.5
A38	*abscessus*	-	A153E	0.0625	0.0625	0.0312	32	2
A39	*massiliense*	-	DEL	0.0156	0.125	0.0625	16	0.0625
A40	*abscessus*	-	Q216R	0.125	0.0312	0.0625	8	4
A49	*abscessus*	-	-	0.125	0.0625	0.0312	16	1
A51	*abscessus*	-	-	0.0312	0.125	0.125	16	1
A54	*abscessus*	-	-	0.0078	0.0078	0.0156	4	1
A58	*abscessus*	-	-	0.0625	0.0625	0.0156	4	0.5
A59	*abscessus*	-	-	0.0625	0.0312	0.0312	8	0.5
A63	*massiliense*	-	DEL	0.0312	0.0625	0.0625	16	0.25
A69	*abscessus*	-	DEL	0.0625	0.0625	0.0312	8	0.0625
A73	*abscessus*	-	-	0.0312	0.0312	0.0078	4	0.5
A79	*abscessus*	-	-	0.0625	0.0625	0.0156	2	0.25
A126	*abscessus*	S91P	DEL	0.0312	0.25	0.125	16	0.0625
A137	*abscessus*	-	-	0.0156	0.0312	0.0078	4	1
A173	*massiliense*	-	DEL	0.125	0.0625	0.0625	32	2
21	*abscessus*	-	-	0.0312	0.0312	0.0078	16	2
A176	*abscessus*	-	-	0.125	0.125	0.0625	128	0.5
A182	*abscessus*	-	-	0.125	0.0312	0.0312	8	0.25
A183	*abscessus*	-	DEL	0.125	0.0312	0.0312	16	0.125
A186	*massiliense*	-	DEL	0.0625	0.0625	0.0312	64	0.0625
A189	*abscessus*	-	-	0.0625	0.0625	0.0312	16	0.5
A197	*abscessus*	-	DEL	0.0312	0.0078	0.002	8	2
A205	*massiliense*	-	DEL	0.0625	0.0078	0.0078	32	0.125
A213	*abscessus*	-	DEL	0.25	0.125	0.0312	64	0.5
A215	*abscessus*	-	-	0.125	0.0625	0.0625	32	0.25
A217	*abscessus*	-	-	0.0312	0.0312	0.0156	8	1
A218	*abscessus*	-	DEL	0.0625	0.125	0.0625	16	2
A222	*massiliense*	-	DEL	0.0078	0.125	0.0625	64	0.0625
A228	*massiliense*	-	DEL	0.0625	0.0312	0.0156	32	0.0625
A232	*abscessus*	-	Q216R	0.125	0.0625	0.0156	32	32
A233	*abscessus*	-	-	0.0312	0.0625	0.0312	8	64
A243	*abscessus*	-	A153E	0.0625	0.0625	0.0625	64	0.5
A244	*abscessus*	-	A170S	0.125	0.0312	0.0312	64	0.125
A247	*massiliense*	-	-	0.25	0.125	0.125	128	0.25
A249	*abscessus*	-	-	0.25	0.0625	0.0312	4	1
A254	*massiliense*	-	-	0.25	0.0625	0.0625	256	64
A266	*abscessus*	-	-	0.125	0.0625	0.0625	16	1
A267	*massiliense*	-	DEL	0.125	0.0312	0.0156	128	64
A268	*massiliense*	-	DEL	0.125	0.0625	0.0156	32	0.0625

A274	*abscessus*	-	-	0.25	0.0625	0.0312	64	1
A289	*massiliense*	-	DEL	0.125	0.125	0.0625	128	0.125
A295	*massiliense*	-	DEL	0.125	0.0312	0.0156	64	256
A297	*abscessus*	-	DEL	0.125	0.0625	0.0312	16	0.0625
A305	*abscessus*	-	-	0.25	0.0625	0.0312	16	1
A311	*abscessus*	-	DEL	0.125	0.125	0.0625	32	8
A312	*abscessus*	-	-	0.125	0.0625	0.0312	16	0.5
A315	*abscessus*	G166R	-	0.5	0.5	0.125	64	4
A317	*abscessus*	-	H8R, E143K	0.125	0.0625	0.0312	16	0.5
A321	*abscessus*	-	-	0.25	0.0312	0.0625	64	0.5
A323	*massiliense*	-	DEL	0.125	0.0625	0.0312	4	0.0625
61	*abscessus*	-	-	0.125	0.0625	0.0625	64	1
A329	*abscessus*	D105G	V6M, H8R, E143K, A218S	0.125	0.0156	0.0312	16	2
A330	*abscessus*	-	DEL	0.125	0.0312	0.0625	8	0.0625
A337	*abscessus*	-	-	0.0625	0.0039	0.0078	4	2
A350	*abscessus*	-	-	0.125	0.0312	0.0312	8	1
129	*abscessus*	-	H8R, E143K	0.125	0.0312	0.0312	4	16
G70	*abscessus*	-	DEL	0.125	0.0625	0.0312	16	2
3	*abscessus*	-	V32I	0.125	0.0156	0.0625	4	1
G72	*abscessus*	-	-	0.125	0.0625	0.125	64	2
G73	*abscessus*	-	G126D, Q216R	0.25	0.0625	0.0625	16	8
G74	*massiliense*	-	DEL	0.0625	0.0312	0.0312	8	4
G75	*massiliense*	-	DEL	0.0625	0.125	0.0625	16	0.0625
G76	*abscessus*	-	-	0.0625	0.0625	0.0625	128	1
G77	*massiliense*	-	DEL	0.125	0.125	0.0625	32	1
G78	*abscessus*	-	Q216R	0.0312	0.0312	0.0312	32	0.0625
G79	*abscessus*	-	-	0.0625	0.0625	0.0625	64	0.0625
2	*abscessus*	-	N2T	0.125	0.0625	0.0625	32	0.0625
G82	*abscessus*	-	-	0.125	0.0625	0.0312	32	0.5
G84	*abscessus*	-	DEL	0.0312	0.0312	0.0312	128	2
G85	*massiliense*	-	DEL	0.0625	0.0156	0.0156	32	0.25
G86	*abscessus*	-	-	0.125	0.125	0.0312	32	0.5
G87	*massiliense*	-	DEL	0.0312	0.25	0.0312	128	0.0625
G88	*massiliense*	-	DEL	0.25	0.125	0.0625	64	0.125
G89	*abscessus*	-	-	0.125	0.125	0.0078	64	0.5
G90	*abscessus*	-	DEL	0.0312	0.125	0.002	32	1
G91	*abscessus*	-	Q216R	0.125	0.125	0.0625	64	2
G93	*abscessus*	-	-	0.0312	0.0039	0.0078	16	1
G94	*abscessus*	-	-	0.0625	0.0625	0.0312	64	2
G95	*massiliense*	-	DEL	0.0625	0.0156	0.0312	32	0.0625
G98	*massiliense*	-	DEL	0.125	0.0625	0.0312	64	0.0625
G99	*abscessus*	-	-	0.0625	0.0625	0.0312	64	2
289	*abscessus*	-	DEL	0.0625	0.0625	0.0625	128	0.0625

G101	*massiliense*	-	DEL	0.0312	0.0625	0.0312	64	0.0625
G102	*abscessus*	-	-	0.125	0.125	0.125	128	1
G103	*abscessus*	-	-	0.25	0.0078	0.0078	2	1
G104	*abscessus*	-	Q216R	0.0625	0.0078	0.0078	128	1
G105	*massiliense*	-	DEL	0.125	0.0156	0.0156	8	0.125
G106	*abscessus*	-	DEL	0.0625	0.0039	0.0039	16	0.125
G107	*massiliense*	-	DEL	0.25	0.25	0.125	32	0.5
G108	*massiliense*	-	DEL	0.0625	0.0625	0.0625	128	0.0625
G109	*abscessus*	-	Q216R	0.125	0.0625	0.125	128	2
G110	*massiliense*	K15N	DEL	4	0.25	0.125	16	1
G111	*abscessus*	-	DEL	0.125	0.0078	0.0156	8	1
G112	*abscessus*	-	DEL	0.25	0.0625	0.0312	64	4
G113	*abscessus*	-	DEL	0.0312	0.0039	0.0156	32	0.5
G114	*abscessus*	-	DEL	0.125	0.0312	0.0625	8	0.125
G115	*abscessus*	-	DEL	0.125	0.0312	0.0312	8	1
G116	*massiliense*	-	DEL	0.0312	0.0039	0.002	4	0.0625
G117	*abscessus*	-	-	0.25	0.0156	0.0078	8	1
G118	*abscessus*	-	-	0.125	0.0156	0.0078	8	1
G119	*abscessus*	-	-	0.125	0.0156	0.0078	8	1
G120	*abscessus*	-	-	0.0625	0.0156	0.0078	16	2
G121	*abscessus*	-	-	0.125	0.0156	0.0078	32	2
G122	*abscessus*	-	DEL	0.0625	0.0039	0.0078	16	4
G123	*abscessus*	-	-	0.25	0.0156	0.0312	8	0.5
G124	*massiliense*	-	DEL	0.0312	0.0078	0.0039	8	0.0625
G125	*abscessus*	-	-	0.125	0.0312	0.0312	8	1
G126	*massiliense*	-	DEL	0.125	0.0312	0.0312	32	0.125
G127	*abscessus*	-	-	0.125	0.0312	0.0312	32	1
G128	*abscessus*	-	-	0.25	0.0625	0.0625	128	2
G129	*abscessus*	-	N2T	0.125	0.0625	0.0625	128	0.25
G132	*abscessus*	-	-	0.125	0.125	0.0312	32	1
G133	*abscessus*	-	DEL	0.125	0.0625	0.0625	32	1
G134	*abscessus*	-	DEL	0.0625	0.0312	0.0078	16	0.125
G135	*massiliense*	-	DEL	0.125	0.0156	0.0156	4	0.125
G136	*abscessus*	-	DEL	0.0625	0.0039	0.0078	64	2
G138	*massiliense*	-	DEL	0.125	0.0156	0.0312	32	0.0625
G139	*abscessus*	-	H8R, E143K	0.0312	0.125	0.0625	64	0.5
G140	*abscessus*	-	H8R, E143K	0.0625	0.125	0.0312	32	2
G141	*massiliense*	-	DEL	0.125	0.125	0.0625	64	0.5
G142	*abscessus*	-	DEL	0.0312	0.125	0.0312	32	0.125
G143	*abscessus*	-	G126D, Q216R	0.0312	0.125	0.0625	64	0.5
G144	*abscessus*	-	N2T	0.0312	0.125	0.0312	16	0.125
G145	*abscessus*	-	-	0.0312	0.0625	0.0312	128	1
G146	*abscessus*	-	-	0.0312	0.0625	0.0625	32	1
G147	*massiliense*	-	DEL	0.0625	0.125	0.0625	32	0.0625
G148	*abscessus*	-	DEL	0.0312	0.002	0.002	16	0.125

G149	*abscessus*	-	DEL	0.0312	0.125	0.0625	32	0.0625
G150	*abscessus*	-	-	0.0625	0.0312	0.0312	16	0.5
G151	*abscessus*	-	-	0.0625	0.0312	0.0312	4	0.5
G152	*abscessus*	-	-	0.0625	0.0312	0.0625	8	2
G153	*massiliense*	-	DEL	0.125	0.0312	0.0312	8	0.125
G155	*abscessus*	-	-	0.125	0.0312	0.0156	128	1
G156	*massiliense*	-	DEL	0.125	0.0625	0.0625	16	0.125
G157	*massiliense*	-	DEL	0.125	0.0625	0.0625	8	0.125
G158	*abscessus*	-	-	0.125	0.0312	0.0312	4	1
G159	*abscessus*	-	-	0.0078	0.002	0.0039	16	0.5
G160	*abscessus*	-	DEL	0.0312	0.002	0.002	32	0.25
G161	*abscessus*	-	DEL	0.125	0.125	0.0625	32	1
G162	*abscessus*	-	DEL	0.0625	0.002	0.0039	8	2
G163	*abscessus*	-	DEL	0.0312	0.002	0.0078	2	0.5
G164	*abscessus*	S77fs[b]	DEL	0.25	0.25	0.125	64	4
G165	*abscessus*	-	DEL	0.0625	0.0078	0.0156	16	0.0625
G169	*abscessus*	-	DEL	0.25	0.0312	0.0625	32	2
G170	*abscessus*	-	DEL	0.0078	0.002	0.002	8	0.125
G172	*abscessus*	-	-	0.25	0.125	0.0625	64	1
G173	*massiliense*	-	DEL	0.25	0.125	0.0625	128	0.125
G174	*abscessus*	-	DEL	0.25	0.0625	0.0625	32	0.125
G175	*abscessus*	-	Q216R	0.25	0.0625	0.0625	32	2
G176	*abscessus*	-	DEL	0.25	0.125	0.0312	8	128
G177	*abscessus*	-	DEL	0.25	0.0625	0.0625	4	2
G178	*abscessus*	-	G126D, Q216R	0.125	0.125	0.0625	128	2
G179	*abscessus*	-	DEL	0.125	0.0625	0.0625	128	0.125
G180	*abscessus*	-	DEL	0.25	0.0625	0.0312	32	128
G181	*abscessus*	-	DEL	0.125	0.0156	0.0625	16	0.125
G183	*abscessus*	-	DEL	0.0625	0.002	0.0312	32	1
G184	*abscessus*	-	-	0.25	0.0312	0.0625	16	0.5
G185	*abscessus*	-	DEL	0.25	0.0625	0.0625	4	0.25
G186	*abscessus*	-	DEL	0.125	0.0156	0.0078	16	0.125
G187	*abscessus*	-	DEL	0.0625	0.0039	0.0312	4	64
G188	*massiliense*	-	DEL	0.125	0.0039	0.0312	8	2
G189	*massiliense*	-	DEL	0.125	0.0039	0.0312	4	128
G190	*massiliense*	-	DEL	0.125	0.0039	0.0625	4	0.0625
G192	*abscessus*	-	-	0.25	0.0039	0.0625	64	128
G193	*abscessus*	-	DEL	0.25	0.0312	0.125	8	0.125
G194	*abscessus*	-	DEL	0.0625	0.0156	0.0625	32	0.0625
G195	*abscessus*	-	DEL	0.125	0.0156	0.0625	32	0.125
G196	*abscessus*	-	-	0.25	0.125	0.0625	32	0.0625
G197	*abscessus*	-	DEL	0.125	0.0625	0.125	32	0.5
G198	*abscessus*	-	DEL	0.0078	0.125	0.0625	64	0.0625
G199	*massiliense*	-	DEL	0.25	0.25	0.0625	64	0.0625
G200	*abscessus*	-	DEL	0.125	0.125	0.0625	32	0.0625
G201	*abscessus*	-	DEL	0.25	0.125	0.0312	64	0.25
G203	*massiliense*	-	DEL	0.125	0.0312	0.0312	8	0.0625

G204	*abscessus*	-	DEL	0.0312	0.0312	0.0625	4	0.5
G205	*abscessus*	-	DEL	0.0078	0.0156	0.0312	8	0.25
G206	*abscessus*	-	-	0.25	0.125	0.0625	32	0.25
G207	*massiliense*	-	H8R, E143K	0.125	0.0156	0.0312	32	0.0625
G208	*abscessus*	-	-	0.125	0.0625	0.0625	128	0.0625
G209	*abscessus*	-	-	0.0312	0.0312	0.0312	128	0.5
G210	*abscessus*	-	DEL	0.125	0.0312	0.0312	8	0.125
G211	*abscessus*	-	-	0.0625	0.0625	0.0625	16	0.125
G212	*massiliense*	-	-	0.125	0.0625	0.0312	64	1
G213	*abscessus*	-	-	0.0625	0.0312	0.0312	64	0.0625
G214	*abscessus*	-	-	0.0625	0.0312	0.0625	128	0.5
G215	*abscessus*	-	H8R	0.0312	0.0312	0.0625	32	0.5
G216	*abscessus*	-	DEL	0.0312	0.0312	0.0156	64	0.0625
G218	*abscessus*	-	DEL	0.0625	0.0625	0.0312	64	0.0625
G219	*abscessus*	-	Q216R	0.125	0.125	0.0625	32	0.125
G220	*massiliense*	-	DEL	0.0312	0.125	0.0312	64	0.0625

[a] DEL, gene deletion.

[b] fs, frameshift.

Supplementary Table 2. FICI of TBAJ-587 combined with frequently used anti-NTM drugs.

	FICI[a] of TBAJ-587 combined with drug indicated							
**Strain**	**CLA[b]**	**IMP**	**AMK**	**RFB**	**LZD**	**CFZ**	**MOX**	**TGC**
M. abscessus ATCC 19977	1	0.75	0.75	1.5	1	1.5	1.5	1
M. massiliense CIP 108297	1.5	1	0.5	1.5	1	1	2	1
M. avium ATCC 25291	1.25	1.5	0.75	2	1.5	1	0.75	1.25
M. intracellulare ATCC 13950	1.25	0.75	1	1.5	0.75	2	1.25	1.25

[a]FICI (fractional inhibitory concentration index) = [(MIC of TBAJ-587 in combination/MIC of TBAJ-587 alone)/(MIC of the second drug in combination/MIC of the second drug alone)]. Synergy, FICI ≤0.5; indifference, FICI ≥0.5 and ≤4; antagonism, FICI >4.

[b]CLA, clarithromycin; IMP, imipenem; AMK, amikacin; RFB, rifabutin; LZD, linezolid; CFZ, clofazimine; MOX, moxifloxacin; TGC, tigecycline.
